# Circumcision in childhood and male sexual function: a blessing or a curse?

**DOI:** 10.1038/s41443-020-00354-y

**Published:** 2020-09-29

**Authors:** Beatriz Bañuelos Marco, Jessica Leigh García Heil

**Affiliations:** 1 Kinderurologie, Charite Universitätsmedizin Berlin, CVK Augustenburger Pl. 1, 13353 Berlin, Germany; 2 London school of hygiene and tropical medicine Keppel St, Bloomsbury, London, WC1E 7HT UK; 3 Desarrollo Cardiológico: Paseo del pago de la Perdiz, 23, Paracuellos de Jarama, Madrid, 28861 Spain

**Keywords:** Quality of life, Urinary tract

## Abstract

Male circumcision (MC) is the first planned surgical procedure ever performed. Nowadays many of these procedures are not necessarily carried out in a medical environment, therefore the real number remains unknown but it is estimated that one third of the men are circumcised. Some authors argue the negative impact of MC on men psychology and sexual life, but objective data are lacking. The purpose of this review is to summarize in the best possible way the literature to clarify this matter. A non-systematic narrative review was performed including articles between 1986 and 2019. The search for literature was carried out between July 2019 to October 2019 and any updates as of March 30, 2020. Although many authors support the hypothesis that circumcision status has an impact on sexual functioning, a negative outcome has not yet been entirely proven. Circumcision might affect how men perceive their body image, and consequently affect their sexual life. We should consider this when analysing the literature about MC and sexual dysfunction, as many of the results are based on specific populations with different attitudes towards this procedure. Sexual function consists of many elements that not only relate to measurable facts such as anatomy, somatosensory and histology. An objective evaluation of the impact of circumcision on sexuality is still challenging, as it affects a wide variety of people that confront sexuality differently due to their sociocultural and historical background. Therefore, individuals can either perceive their circumcision status as a blessing or a curse depending on the values and preferences of the different communities or social environments where they belong.

## Introduction

Male circumcision (MC) is the oldest planned surgical procedure performed around the world [1]. Although it is estimated that one third of males underwent circumcision, the true number might be higher as we can recall due to the nature of how it is carried out. Many of these are performed in places other than primary centres or clinics, like in the private homes or the physician’s clinic [2].

The age of circumcision varies depending on the country and the sociocultural and religious background of the community. Whereas in non-muslim developing countries MC it is part of coming of age process or ceremonies, for many other countries MC occurs most commonly during infancy. Most of the world’s male population is uncircumcised, while in the United States due to the policy by the American Academy of Pediatrics (AAP) there is tendency towards neonatal circumcision [2].

The recent AAP guideline on MC reversed its prior stand, now stating that the “health benefits of newborn MC outweigh the risks,” allowing access to the procedure if the parents agree to do so [3]. However, neonatal circumcision continues to be a polemic issue. Although from the medical point of view MC brings many benefits, these are not necessarily extended to all children, therefore controversial as an only reason to perform neonatal circumcision. Around 1% of the patients would experience complications, either acute or later in life due to cosmetic or medical reasons [4]. A negative psychological and sexual impact of circumcision has been argued, but solid, scientific data are lacking.

The purpose of this article is to present a non-systematic narrative review based on the current knowledge of the impact of childhood or neonatal circumcision on male sexual function.

## Literature review

### Search strategy and study selection

A non-systematic narrative review was performed using the following search terms: “circumcision”; “children circumcision”; “neonatal circumcision”; “circumcision and sexual dysfunction“; “premature ejaculation (PE)”; and “erectile dysfunction and circumcision”. The search for literature was performed between July 2019 and October 2019 through the electronic databases: PubMed (MEDLINE), WHO/UNAIDS, Google-Scholar, Semantic-Scholar and Research Gate and included articles between 1986 and 2019. After the first revision of the article any updates as of March 30th 2020 were added.

Citations and abstracts identified by the search strategy were screened. There were no languages or publication year limitations. Those studies lacking data were not included due to possible limitations or bias. There was no exclusion due to cultural/background differences of the population studied.

Randomized controlled trials, meta-analyses, systematic reviews and large studies were the preferable choice if available. Internet searches were performed if necessary to clarify terms or to search relevant information concerning policies.

A resume table follows each section including the articles reviewed, aims of the studies, number of patients and outcomes.

### Circumcision and PE

PE [5] is one of the most common sexual dysfunction in all men around the world. It is defined by ejaculation occurring within less than 1 min after penetration and its aetiology remains unknown. Many studies have argued a relation between circumcision and PE. One of the hypotheses suggests that PE might be caused by somatic and neurobiological disorders. Evidence suggests a relationship between the higher amount of nerve fibres in redundant prepuce and high sensitivity as a possible cause of PE [6]. In another prospective study post-circumcision penile mucosal cuff length, scar thickness and PE syndrome were evaluated and no association was found when compared to the healthy control group [7].

Most of the studies were carried out in adult population, therefore the impact of MC during infancy in PE it is difficult to evaluate. Based on the results of these studies it is not possible to conclude that circumcision has a correlation with PE as there is not enough evidence.

In the prospective study performed by Gao [8] on 998 men, those who had been circumcised reported better ejaculatory control and PE severity than those uncircumcised at 3-, 6-, 9- and 12-months follow-up. In the study performed by Alp et al. [9] the intravaginal ejaculation latency time (IELT) was evaluated. The mean IELT was 104.36- ±66.21 s and 123.56 ± 54.44 s before and after circumcision, respectively. For the individual evaluation of PE, the PE diagnostic tool (PEDT) scores included the following items: perceived control, frequency, minimal stimulation, distress and interpersonal difficulty. The mean scores were 4.26 ± 2.91 before and 2.63 ± 1.82 after circumcision. They concluded that improvements in IELT and PEDT after circumcision were statistically significant.

In the systematic review and metanalysis conducted by Yang [10] circumcision status was not associated with PE (OR, 0.90; 95% CI, 0.72–1.13; *p* = 0.37). Most of the studies and systematic reviews support the evidence favouring a lower prevalence of PE in circumcised males [9, 10, 11].

Whereas Tang and Khoo [11] showed that 50.9% of circumcised men and 28.9% of uncircumcised men suffered from PE (OR, 2.56; 95% CI, 1.44–4.55; *p* = 0.001), other studies which established that circumcision is associated with PE like Richters et al. evaluate as PE the facility of coming to an orgasm [12]. It happened on 26% of circumcision men and 22% of control group (OR, 1.28; 95% CI, 1.15–1.42; *p* < 0.0001). However, the difference was not statistically significant after adjustment (*p* = 0.11). Frisch et al. [13] conducted a cross-sectional study in Denmark. The proportion of PE among circumcised men and uncircumcised group was 59% and 61%, respectively, (OR, 0.92 95% CI 0.60–1.41). In the case control study from Aydogmus et al. [14] PE was observed in 32% of the patients in the preoperative study group, 22% of the postoperative and 23% of controls. Their results showed no significant difference between any of these groups for PEDT and IELT scores, neither for PE. However, a significant improvement was seen when pre and postoperative values were compared for the three items.

The studies related to “PE and circumcision” are summarized in Table 1.

**Table 1:** Male circumcision and premature ejaculation.

Author	Year	Type	Number of patients/studies	Aim	Outcomes
Tian et al. [45]	2013	Systematic review and meta-analysis	18740 patients/ 10 studies	Assess sexual function after male circumcision	Circumcision is unlikely to affect male sexual function Lack of evidence in the current literature
Malkoc et al.	2012	Prospective study	20 volunteers	Determine free nerve ending in foreskin and the effects on premature ejaculation	No statistic correlation between the FNE and premature ejaculation
Alp et al.	2014	Prospective study	30 volunteers	Effect of adult circumcision on ejaculation parameters and evaluate relationship between intravaginal ejaculatory latency time and PEDT	No correlation between ejaculation time and PEDT scores improvement of PE and IELT after circumcision
Yang et al.	2018	Systematic review and meta-analysis	12 studies 21,589 men	Effects on premature ejaculation	Circumcision has no effect on PE (OR, 0.90) and orgasm (OR 1.04)
Gao et al.	2015	Prospective study	1198 men	Adult male circumcision and pE	Findings suggest positive effect on IELT, sexual satisfaction, PE
Tarhan et al.	2013	Prospective study	10,173 men	Relationship between post-circumcision penile mucosal cuff length, circumcision scar thickness and the PE syndromes	No relationship was observed
Richters et al.	2006	Telephone survey	10,173 men	Assess prevalence of circumcision and study the impact on sexual life, transmitted diseases, etc	Not associated with either protective or harmful sexual health outcomes
Tang et al.	2011	Cross-sectional study	2017 men recruited, 93% response	Prevalence of PE and associated factors	Factors associated with PE were: circumcision Indian ethnicity Intercourse frequency <5 times a month
Frisch et al.	2011	Survey	5552 (men and spouses)	Examine consequences of male circumcision	Circumcision is associated with frequent orgasm difficulties in Danish men
Aydogmus et al.	2016	Case control prospective study	37 cases and 30 controls	Study of psychological and sexual effects in adult men and its changes following circumcision	Social anxiety and anxiety levels decreased after circumcision in Turkish men

*PE* premature ejaculation, *PEDT* premature ejaculation diagnostic tool, *IELT* intra ejaculatory latency time, *FNE* free nerve ending, *OR* odds ratio.

### Circumcision and male sexual dysfunction

Sexual dysfunction it is defined in the DSM V as an inability for sexual reaction and satisfaction responses during sexual activity. It includes disorders of sexual desire or interest, arousal, orgasm and sexual pain [15].

According to Barlow’s model sexual dysfunction is based on cognitive interference and anxiety [16]. The role of attention and its influence on sexual dysfunction has been studied by Jong [17] and his research suggests that sexual arousal could be facilitated or disrupted by cognitive bias. Thus, an individual suffering a negative self-perception is prompt to drive his/her attention away from the erotic stimuli. This is likely to result in an autonomic nervous system response and consequently creates a negative feedback loop affecting the sexual function.

The attitude of men towards their own circumcision was shown to be more important in the study carried out by Bossio [18], which explores the relationship between attitude towards one’s circumcision status, timing of one’s circumcision and sexual correlates. After studying 811 men (367 circumcised as newborns, 107 during childhood, 47 as adults and 290 uncirumised) they concluded that negative attitude towards one’s circumcision is related to worse body image and sexual functioning, and not the circumcision status of men.

Few studies have investigated the effect of circumcision on body image and social phobia [14]. In the previous mentioned study from Aydogmus et al. [14] self-body image and social anxiety were studied through Body Cathexis Scale (BCS), Liebowitz Social Anxiety Scale (LSAS). After circumcision a significant reduction was seen for both BCS and LSAS. This study was carried out among Turkish men, however the conclusions showed the importance of social anxiety and the correspondingly negative effects of it on sexual function and satisfaction. The sample described in their study did not experience any worsening of their PEDT scores, IELT values or PE. In addition, when evaluating BCS and LSAS items on sexuality, circumcision improved sexual satisfaction in the studied group. Therefore, this study supports the evidence that men’s attitude towards circumcision is a determinant factor for sexual satisfaction, whereas its negative impact in PE or PEDT scores is not yet consistently proven.

### Circumcision in the infancy and male sexual dysfunction

The possible effect of infant circumcision on male sexual dysfunction cannot be determined directly and it is still in debate. It can be inferred from adult male sexual function.

Hypothetically childhood circumcision could lead to histological changes such as keratinization of the glans surface, diminishing sensitivity and consequently sexual excitability. It has also been suggested that lesions of the prepuce could cause atrophy of the brain circuitry leading to lower excitability [19].

However, a recent systematic review of histological and anatomical data on sensory receptors in the penis, which includes also those changes that appear in puberty concludes that nerve endings related to sexual pleasure reside in the glans and the foreskin have no role in sexual sensation [20]. Meissner corpuscles of the foreskin had unlikely a role in erotic sensation as their density decreases at the age when sexual activity gets higher [20]. Free nerve endings showed no statistic correlation with sexual response and tactile sensitivity of the glans, as the later decreases with sexual arousal and it is not linked to sexual sensation. Hypotheses on higher penile sexual sensation after circumcision correlate to the bigger surface accessible of genital corpuscles after the removal of the foreskin, based on their distribution in the glans. Furthermore, vibrational and thermal sensitivity during intercourse are perceived as a reward mechanism and neither of them is related to circumcision status. In the prospective study carried out by Aydur [21] they reported no relationship between circumcision age and overall male sexual function. When comparing subgroups of specific spheres of sexual function, avoidance and communication appeared to be influenced by age at the time of circumision. However, these seem to be very subjective matters related to negative body image perception as previously described. Many studies have proven that MC has no negative effect on sexual function. The RCTs from sub-Saharan Africa carried out by Kigozi [22], showed no statistically significant difference on sexual function after including more than 10,000 men. Aydur et al. [21] could not find any association between age of childhood circumcision and overall sexual function in men between 22 and 44 years old, however as men in Turkey are mostly circumcised in childhood there was no uncircumcised men control group. Morris’ systematic review carried out in Australia [23] on early MC, with a total of 40,473 men, showed that medical circumcision (MC) does not adversely affect sexual function, sensitivity or pleasure. In the Danish study from Shabanzadeh et al. [24] no statistical difference could be found in the outcomes after undergoing non MC, however problems in reaching orgasm increased and ED was reported inconsistently. This systematic review contained 38 studies including two randomised trials. The only differences that they could find in sexual function for circumcised men were decreased PE and increased penile sensitivity. Those men who were circumcised younger were less prone to suffer sexual dysfunction than those circumcised later in life.

There is enough literature supporting the fact that childhood circumcision has no negative influence in sexual function per se. A survey carried out in the United Kingdom (AIDS 2015) confirmed these findings also on 6293 men and 8869 women [25].

The studies related to “circumcision in the infancy and male sexual dysfunction” are summarized in Table 2.

**Table 2:** Circumcision and male sexual function.

Author	Year	Type	Number of patients/studies	Aim	Outcomes
De Jong et al.	2009	Review	n/a	Role of attention in sexual arousal	Attention focus might be essential in the voluntary control of sexual arousal and its achievement, through sexual fantasy or nonsexual cognitions
Bossio et al.	2018	Retrospective study	811 men	Exploring the impact of individual attitude towards circumcision, timing of it and sexual correlates	Lower satisfaction with one’s circumcision status relates with worse body image and thus sexual dysfunction
Taylor et al.	1996	n/a	22 autopsies	Assess type of tissue missing from adult circumcised penis	Circumcision also ablates junctional mucosa. Non consistent result
Cox et al.	2015	4	41 studies	To examine histological correlates relevant to penile sensitivity and sexual pleasure	Sexual response is unlikely to involve Meissner’s corpuscles whose density in the prepuce disminishes at the time of life when male sexual activity is increasing. FNE show no correlation with sexual response. Tactile sensitivity of the glans decreases with sexual arousal and it is unrelated to sexual sensation. Thermal sensitivity seems part of the reward mechanism of intercourse. Vibration sensitivity is not related to circumcision status
Aydur et al.	2007	Prospective study	107 men	Determine relationship between circumcision age and sexual function	Childhood circumcision might affect some domains but not overall male sexual function
Kigozi et al.	2008	Randomized control trial	4456 subjects	Investigate the relationship between adult male circumcision and sexual satisfaction and function in men	Adult male circumcision does not adversely affect sexual satisfaction or clinically significant function in men
Morris et al.	2013	Systematic review		Examine: imairing or improvement of sexual function after circumcision	No adverse effect on sexual function proven
Shabanzadeh et al.	2016	Systematic review	38 studies	Determine if circumcision medical indication or age at circumcision had an impact on perceived sexual function in males	Inferior male sexual function following circumcision could not be supported
Homfray et al.	2015	Stratified probability survey	6293	Association between circumcision and sexual function	No statistic association between circumcision and poorer sexual function (OR 0.95; 95% IC 0.76–1.18)

*n/a* no applicable, *OR* odds ratio, *IC* interval of confidence.

### Age of circumcision

There is still a controversial discussion with arguments in favour of early MC over delayed and elective MC at an older age. Using the Golombok-Rust Inventory of Sexual Satisfaction (GRISS) Aydin et al. conducted a prospective study [26] with the objective of analysing sexual dysfunction among circumcised men in different infancy and childhood age groups. Prevalence of sexual dysfunction in overall and specific areas of GRISS showed no statistically significant difference among the three groups. The 3–5 years group showed worse results on the scores of coitus frequency, degree of satisfaction, sensuality and erectile function but no statistically significant differences were found. The 3–5 years age group had the best mean score of ejaculatory function. Only in the avoidance domain was it possible to find statistically significant differences between groups, with lower avoidance in the infantile circumcision group compared to those who underwent circumcision at the age of 3–5 years (*P* < 0.05). After dividing men into sexually functional and dysfunctional groups and comparing with mean age at the time of circumcision there was no difference in overall sexual function. However, in the communication domain those men sexually dysfunctional were statistically significantly younger.

The study of Armagan [27] evaluated the possible negative impact of circumcision during the phallic period (3- to 6-year-old) on psychosexual functions in 302 adult males. No statistical difference was found between the mean total IIEF scores (group-1, phallic period: 25.1 ± 4.8, group-2, non-phallic period: 25.4 ± 4.6, *P* > 0.005); overall satisfaction was also found to be comparable. The PEDT scores were comparable between both groups (group-1: 8.2 ± 4.8, group- 2: 8.7 ± 5.4, *P* > 0.05).

To evaluate psychological status Beck depression scores were performed, which showed no differences between groups (group-1: 10.8 ± 10.4, group-2: 9.8 ± 8.9, *P* > 0.05). Their results supported that circumcision during the phallic period has no correlation with psychosexual dysfunction in the adulthood.

Cuceloglu [26] reported that circumcision performed after 7 years of age could negatively affect PE compared to those who underwent the procedure at an earlier age. The review by Yavuz et al. hypothesized that as circumcision is a part of the community rituals or practices, the child not only won’t perceive it as a threat, but also he would be aware of its significance and the consequences that implies [28]. They advise to perform circumcision at an age when he can make his own decisions. Nevertheless, it needs to be remarked that this study was carried out in a population where circumcision is part of the sociocultural background, thus having a very important role and influence in the perception on body image and sexuality.

On the somatosensory level, Bleustein et al. [29] carried out a study using a battery of quantitative somatosensory tests evaluating small to large axon function. After performing a comparative analysis of uncircumcised and neonatally circumcised men, their results showed no significantly differences in the quantitative somatosensory tests of the glans.

The studies related to “age of circumcision” are summarized in Table 3.

**Table 3:** Age of circumcision and male sexual function.

Author	Year	Type	Number of patients/studies	Aim	Outcomes
Dave et al.	2018	Guideline systematic review	229 studies	Present evidence on benefits of circumcision	
Tian et al. [45]	2013	Systematic review and meta- analysis	18740 patients/10 studies	Assess sexual function after male circumcision	Circumcision is unlikely to affect male sexual function Lack of evidence in the current literature
Kigozi et al.	2008	Randomized trial	4456 men	Relationship between male circumcision and sexual satisfaction/function	Adult circumcision does not affect sexual function adversely
Armagan	2014	Prospective study	302 men	Examine if circumcision during phallic period (3–6 years) has a negative impact on psychosexual function in adults	No statistical difference between the mean total IIEF scores(group 1:25.1 ± 4.8 group 2: 25.4 ± 4.6, *p* > 0.05) neither in subdomains (erectile function, orgasm, sexual desire, intercourse satisfaction, overall satisfaction group 1: 8.2 ± 4.8, (group 2: 8.7 ± 5.4, *p* > 0.05) Beck depression scores were also comparable(group 1:10.8 ± 10.4 group 2: 9.8 ± 8.9, *p* > 0.05)
Cuceloglu et al.	2012	Case control	40 cases/40 control	Analyze effect of age at circumcision on PE	Age at circumcision >7 years old was associated with PE
Bleustein et al.	2005	Comparative anlysis	125 patients	Evaluation of penile sensory thresholds in circumcised men during newborn period	Circumcision is not quantitatively related with the results of somatosensory testing at glans
Payne et al.	2007	Comparative study	20/20	Evaluate genital and non genital sensation, as function of sexual arousal in circumcised men	No differences between circumcised and uncircumcised men

*PE* premature ejaculation, *IIEF* international index of erectile function.

### Medical benefits of circumcision during childhood

Circumcision is a common procedure performed historically and culturally because of its medical benefits, among many other reasons. Despite its advantages it is still controversial if the benefits outweigh the risks, and if so when it would be the better timing for it. Although there are many arguments in favour of MC, it seems reasonable to question if infant MC offers a true benefit over MC later in life, when the boy can decide for himself. There are few studies comparing the merits of MC at different ages. On the systematic review of the literature by Morris et al. [30], they studied infant MC vs MC in childhood, adolescence and adulthood. They emphasized the benefits of an early circumcision to avoid losing the medical benefits of it.

In the first year of life, UTI in males is most common and affects 1–2% of uncircumcised boys compared to 0.1–0.2% of boys who are circumcised [31]. In the study performed by Ellison [32] 5560 boys with uretero-hydronephrosis (UH) and 11,120 healthy boys were identified through a nationwide database and after the multivariate analysis newborn circumcision was associated with a significantly decreased risk of UTI (OR 0.36 [95% confidence interval (CI) 0.29–0.44] for boys with hydronephrosis; OR 0.32 [95% CI 0.21–0.48] for healthy patients). The necessary number to treat to avoid one UTI was 10 for those children with UH compared with 83 in healthy boys.

Other medical benefits include reducing the risk of balanitis [30] and it also eliminates the risk of suffering lichen sclerosis, which is diagnosed in up to 40% of foreskins removed for phimosis [33]. Several STIs are also most common in uncircumcised male, including human papillomavirus (HPV) [34] Trichomonas vaginalis [4], Mycoplasma genitalium, [30] syphilis [34] and HIV [35]. Reduced risk of penile cancer and cervical cancer, the latter due to the reduction in HPV transmission are also arguments of many physicians to defend newborn MC as a public health policy [1, 4, 31].

On the other hand, the prevalence of adverse events of MC, in the literature review by the American Association of Pediatry and the Center of Disease Control research of 1.4 million MCs from 2001 to 2010 (93% in newborns) [36], showed that in less than 0.5% of newborn infants there is a complication and these are mostly minor. Meatal stenosis appears in 0.01–1% of males during the follow-up although it is not necessarily related to the procedure itself but to foreskin diseases or as an accidental finding [2].

The studies related to “medical benefits of circumcision during childhood” are summarized in Table 4.

**Table 4:** Medical benefits of circumcision during childhood.

Author	Year	Type	Number of patients/studies	Aim	Outcomes
Dave et al.	2018	Guideline systematic review	233 studies	Present evidence on benefits of circumcision	For the Canadian neonates, the protective benefits MC are not comprehensive, may not last over a lifetime and can be achieved by other preventive health measures
Abara et al.	2017	Literature review	n/a	Review the state, function and care of prepuce and the current practice of childhood male circumcision Focus on newborn male circumcision	Circumcision is safe More studies are need to claim that benefits outweigh the risks
Lerman et al.	2001	Review	n/a	Present evidence on benefits of circumcision	Lack of evidence 80 circumcisions needed to prevent 1 UTI Not proven sexual dysfunction
Morris et al.	2012	Review	n/a	Analysing medical benefits of infant circumcision versus circumcision in later childhood, adolescence and adulthood	Infant circumcision is safe, simple, convenient and cost-effective They conclude that infancy is the optimal time for circumcision
Morris et al.	2017	Systematic review	153 studies	Determine if the USA policies on male circumcision apply comparably to Anglophone countries: Australia and New Zealand	Early infant circumcision will improve public health by reducing prevalence from highly prevalent foreskin-related conditions and diseases
Ellison et al.	2018	Prospective study	5561	Evaluate if boys with an early diagnosis of hydronephrosis who undergo newborn circumcision will have reduced rates of UTI	NPT: 10 (With hydronephrosis reduced risk of UTI for those with isolated hydronephrosis (OR 0.35, 95% CI 0.26–0.46) ureterpelvic juntion obstruction (OR 0.35, 95% CI 0.20–0.61)
Morris et al.	2017	Systematic review	108 studies	Relation between penile inflammatory skin condition and penile cancer. Evaluate the protective effect of circumcision	68% lower prevalence of balanitis in circumcised male Balanitis increases 3.8 the risk of penile cancer
Tobian et al.	2009	Randomized control trial	5534 subjects	Assess efficacy of circumcision in prevention of HSV-2, HPV infections and syphilis in HIV negative subjects between 15 and 49 years old	At 24 months HSV-2 seroconversion was 7.8% in the intervention group and 10.3% in the control group (HR, 0.72; 95% confidence interval [CI], 0.56–0.92; *P* = 0.008) HPV prevalence 18.0% in the intervention group and 27.9% in the control group (RR 0.65; 95% CI, 0.46–0.90; *P* = 0.009). syphilis, no differences (HR, 1.10; 95% CI, 0.75–1.65; *P* = 0.44)
Bailey et al.	2007	RCT	2784 subjects	Determine the protective effect of male circumcision on HIV-1 infections Age 18–24. Kenia Follow-up 24 months	Protective effect 60% 2-year incidence of HIC 2.1% (CI 1.3–3.0) in circumcised and 4.2% (3.0–5.4) in control group (*p* = 0.0065) RR 0.47 (0.28–0.78)
El Bcheraoui et al.	2004	Retrospective cohort study	1,400,920 circumcised male 93.3% as newborns 41 Adverse events (AE) studied	Estimate the incidence rate of MC-associated AEs and to assess whether AE rates differed by age at circumcision	Incidence of total AE less than 0.5% AE were 20-fold and 10-fold higher for those 1–9 and 10 years or older respectively, when compared to those younger than 1 year

*n/a* not applicable, *MC* male circumcision, *UTI* urinary tract infection, *IC* interval of confidence, *HR* hazard ratio.

## Discussion

The impact of childhood circumcision in the sexual lives of men is still very difficult to evaluate and fully understand. Sexual satisfaction comprehends a wide range of assets not only anatomical or physiological, but also personal attitude towards genital image and full body perception, individual psychology, previous experiences before the moment of the evaluation, partnership status, religion, cultural background and perception of sexuality in their sociocultural background.

The reduction of the risk for many diseases is on of the main arguments on favour of infant circumcision. It is proven in many studies that MC reduces the risk of lower UTI, penile cancer and several STI. Also, it is important to address that the benefit of it is higher for those children with urological conditions, such as UH, vesicoureteral reflux and ureteropelvic junction obstruction [4, 5]. Nevertheless, for the majority of uncircumcised children those medical benefits are also achievable through proper hygiene measures [6]. The reduction of sexual transmitted diseases (STDs) should be based on sexual education and therefore safe sexual practices should be encouraged and not substituted by the benefits of circumcision. However, we must not forget that where sexual education and low-risk sexual behaviour is not a priority or not available for all the individuals, the impact of circumcision as a prevention measure against HIV and other STDs is relevant.

MC as any other surgical procedure presents potential harms, 1% of the patients would present acute complications or/and problems related to it in the follow-up. The American Academy of Paediatrics and the American Academy of Family Physicians recommended access to circumcision concluding that the benefits outweigh the risks. However, they do not endorse routine neonatal circumcision [37].

The impact of MC on male sexual function is still difficult to infer. The differences between populations and their cultural background are a reason for the discrepancies found in the literature. The survey carried out in Denmark by Frisch in 2011 [13] showed that circumcised men were more likely to experience difficulties reaching their orgasms, while Gao study in 2015 [8] had opposite results, showing that after circumcision the IELT was longer, and also the scores of control over ejaculation, satisfaction with sexual intercourse and severity of PE were better than men before circumcision.

Although it may seem that this controversies make impossible to reach a consensus there is consistent data supporting that no objective histological or physiological reasons lead to an increased risk on sexual dysfunction, as there is also data on adults showing that not only it does not adversely impact functioning e.g., Kigozi et al.; Laumann, Masi, Zuckerman, 1997; Payne et al. but it might improve it (e.g., Senel et al.) ([22, 38–41]). However, to complicate this challenging topic some studies reported decreased sexual function after circumcision (e.g., Fink et al.; Kim and Pang; Shen et al. [42–44].

Still, sexual function is difficult to test mainly based on score scales or IELT as it should also include an evaluation for individual sexuality satisfaction, which is constituted by different items, many of them difficult to measure and compare. The interesting study performed by Bossio explores the relationship between men’s attitude towards circumcision, its timing and sexual correlates. They confirmed the importance of negative attitude towards one’s circumcision as it leads to a worse body image perception influencing sexual functioning [18]. Men suffering sexual dysfunction experience a lack of control and focus related to their performance demands (internal or external) diverting the attention from the erotic stimuli and therefore leading to non-satisfactory experiences. This as already explained previously causes a negative feedback loop, which is detrimental to sexual response.

Psychoanalitical theories considered the preputium as part of the penis and its removal might cause the perception of a deficiency. Nevertheless, the studies data are not consistent enough and present a high bias because of the population where it was carried out.

The varying opinions may be related to different cultures, religions and even social trends or preferences of the study population, as well as that of investigators. It has been reported that among Turkish population the absence of circumcision is perceived negatively, boys felt ashamed of it and they are not comfortable with their body image. Another study performed on Turkish population reported that circumcision created a social stress and children did not feel themselves as part of the community. Apart from Muslims, it was reported that Jewish children also considered circumcision as a positive event and culturally relevant and did not feel it as a threat or punishment.

Some groups oppose to circumcision, as they believe that performing routinely non-medical circumcision in infants is similar to carry out a surgery without an informed consent, as at the moment when it is performed neither the child can consent, nor there is a pathology to be treated, just a theoretical proven benefit. This might seem as a strong statement, but physicians must acknowledge that invasive procedures should be only performed when a medical benefit is expected. The decision whether or not to circumcise remains as a challenge not only for the parents but for the clinicians who must inform them.

Therefore, the environment where children mature and develop their sexuality has a consequent influence on their perception of circumcision. Sociocultural background, religion and education are factors playing a very important role in the individual’s attitude towards circumcision and also on the perception of body image and self-esteem. Historical and cultural heritage also influence how sexuality appears in several dimensions in the lives of individuals. Personal beliefs and preferences, social trends and pressures on the parents also influence in experiencing it as a bad experience or as a ritual that creates an important bonding moment with their own community. Therefore, individuals can either perceive their circumcision status as a blessing or a curse depending on the values and preferences of the different communities or social environments where they belong.

### Limitations

This review presents several limitations, being a non-systematic narrative review only a limited number of databases and studies were screened. There were no limitations on the cultural background of the studies, which can relatively lead to several bias. Firstly, the inclusion of men who are unsatisfied with their circumcision results in mixed data. On the other hand, it is frequent to find participant bias in studies including men who undergo electively circumcision as adults and are based on self-report measures, which are not entirely objective, thus those men are likely to report better results.

## Conclusion

There is a need to explore men’s attitudes towards their circumcision status and how these might impact men’s body image and sexual functioning. Current research suggests that circumcision status may be related to sexual functioning. The nature of this relationship is unclear as it can also be affected by many factors previously discussed that are difficultly measured due to their subjective or environmental complex nature; compared to the more objective anatomical, somatosensorial or histological elements that may have an effect on this relationship.

The impact of circumcision on the complex and dynamic development of sexuality is difficult to classify because it comprehends very different groups of people with a wide variety of ways of perceiving not only sexual satisfaction, but also their own exploration of sexuality due to their sociocultural and historical background. More than trying to define it as a curse or a blessing, the focus should be on the proven medical benefits and the demystification of its negative impact on functional sexuality. Parents could then make an informed decision based on objective facts and reduce any negative perception by the children towards their own circumcision as much as possible.
