# Correction to: Circumcision in childhood and male sexual function: a blessing or a curse?

**DOI:** 10.1038/s41443-021-00501-z

**Published:** 2021-11-29

**Authors:** Beatriz Bañuelos Marco, Jessica Leigh García Heil

**Affiliations:** 1 Kinderurologie, Charite Universitätsmedizin Berlin, CVK Augustenburger Pl. 1, 13353 Berlin, Germany; 2 London school of hygiene and tropical medicine Keppel St, Bloomsbury, London, WC1E 7HT UK; 3 Desarrollo Cardiológico: Paseo del pago de la Perdiz, 23, Paracuellos de Jarama, Madrid, 28861 Spain

**Keywords:** Quality of life, Urinary tract

Correction to: *International Journal of Impotence Research* 33:139–148; 10.1038/s41443-020-00354-y, Published online 29 September 2020

The article Circumcision in childhood and male sexual function: a blessing or a curse?, written by Beatriz Bañuelos Marco, Jessica Leigh García Heil, was originally published Online First without Open Access. After publication in volume 33, issue 2, page 139–148 the author decided to opt for Open Choice and to make the article an Open Access publication. Therefore, the copyright of the article has been changed to © The Author(s) 2020 and the article is forthwith distributed under the terms of the Creative Commons Attribution 4.0 International License, which permits use, sharing, adaptation, distribution and reproduction in any medium or format, as long as you give appropriate credit to the original author(s) and the source, provide a link to the Creative Commons licence, and indicate if changes were made. The images or other third party material in this article are included in the article’s Creative Commons licence, unless indicated otherwise in a credit line to the material. If material is not included in the article’s Creative Commons licence and your intended use is not permitted by statutory regulation or exceeds the permitted use, you will need to obtain permission directly from the copyright holder. To view a copy of this licence, visit http://creativecommons.org/licenses/by/4.0.

Open access funding enabled and organized by Projekt DEAL.

